# Genomic Characteristics of a Chicken Infectious Anemia Virus in Contaminated Attenuated Vaccine

**DOI:** 10.3389/fvets.2022.925935

**Published:** 2022-06-16

**Authors:** Longfei Chen, Qi Su, Yan Li, Jinjin Wang, Yawen Zhang, Shuang Chang, Yixin Wang, Peng Zhao

**Affiliations:** ^1^College of Veterinary Medicine, Shandong Agricultural University, Tai'an, China; ^2^Shandong Provincial Key Laboratory of Animal Biotechnology and Disease Control and Prevention, Tai'an, China; ^3^Shandong Provincial Engineering Technology Research Center of Animal Disease Control and Prevention, Tai'an, China

**Keywords:** chicken infectious anemia virus, recombinase-aided amplification assay, contamination, genome analysis, live vaccines

## Abstract

Chicken infectious anemia virus (CIAV) can be transmitted by contaminated live vaccines, and causes huge economic losses. This study evaluated the contamination status of CIAV in 24 batches of vaccines by recombinase-aided amplification assay (RAA), fluorescence quantitative PCR and dot blot assay, and then found a contaminated attenuated vaccine. The whole genome of the CIAV contaminant was then sequenced and named JS2020-PFV (Genbank accession number: MW234428, 2296bp). It showed 94.5 to 99.9% identities with reference strains and shared the closest evolution relationship with AB1K strain which was isolated from a chicken farm in Turkey. All of these suggested that the use of CIAV contaminated live vaccine may be one of the reason for its epidemic in poultry.

## Introduction

Chicken infectious anemia virus (CIAV) induces severe anemia and immunosuppression in poultry (1). After its first report in 1979 (2), CIAV has been detected worldwide and has caused huge economic losses (3). Recently, CIAV infections have also frequently appeared in different chicken farms in China (4–8). The pathogenesis of CIAV has been well understood that it mainly causes the atrophy of bone marrow, hematopoietic tissue, and lymphatic tissues (e.g., thymus) in young chickens (9). CIAV can be transmitted both vertically and horizontally via respiratory and digestive tracts (1), but how it spreads across different regions and even countries remains unclear.

It is worth noting that the first strain of CIAV was isolated from the contaminated attenuated vaccine (2). And, after that, similar reports emerged in a lot of countries (10–12), revealing that the use of contaminated live vaccines is an important way of the transmission of CIAV. Through interactions with attenuated vaccine strains or co-infected viruses, CIAV triggers more serious clinical symptoms with a strong immunosuppressive ability (13–15). More importantly, the attenuated vaccines are usually inoculated at a low age, the CIAV infection caused by it will greatly interfere with the immune responses against vaccines, resulting in a secondary infection (16, 17). Therefore, monitoring the vaccine contaminations is of great significance for preventing CIAV infection in poultry.

This study aims to screen the potential CIAV contamination in live vaccines by recombinase-aided amplification (RAA), fluorescence quantitative PCR assay (qPCR) and dot blot. The whole genome of the contaminated CIAV strain was sequenced for further analysis.

## Materials and Methods

### Vaccine Collection

Twenty-four batches of live vaccines against Newcastle disease virus (*n* = 6), infectious bronchitis (*n* = 5), infectious laryngotracheitis encephalomyelitis (*n* = 4), fowl pox (*n* = 4), Marek disease virus (*n* = 3), mycoplasma gallisepticum (*n* = 1), viral arthritis (*n* = 1), were collected from a layer chicken farm in Heibei province from Jun 2020 to Jun 2021. All these vaccines were selected randomly from the same batch of vaccines purchased by this chicken farm before vaccination for detecting potential exogenous virus contaminations. All vaccines were not opened before testing. All these vaccines were diluted with normal saline according to the instructions, and 200 μl of the dilute was used for DNA extraction.

### DNA Extraction

DNA was extracted using the DNA extraction kit (Omega, Bio-Tek, USA) according to the manufacturer's instructions as the template for RAA, qPCR and dot blot.

### RAA

The primers and probe were designed according to the published CIAV genome (Tables 1, 2). The RAA fluorescence kit and RAA amplification instrument were provided by Hangzhou ZC Bio-Sci & Tech Co., Ltd. (Hangzhou, China). ddH_2_O was used as the negative control and standard plasmid containing full-length CIAV genomes was constructed by our lab and used as a positive control (7). RAA detection methods and primer probes are shown in Table 1.

**Table 1:** Sequence of primers used in this study.

**Primers**	**Sequence**	**Product length**
C-F	CAGTAGGTATACGCAAGGCGGTCCGGGTG	277bp
C-R	CACACAGCGATAGAGTGATTGTAATTCCAG	
C-probe	CAAGTAATTTCAAATGAACGCTCTCCAAGA [FAM-dT]A[THF][BHQ1-dT]CCACCCGGACCATCAAC	
CAV-com-F1	GCATTCCGAGTGGTTACTATTCC	842bp
CAV-com-R1	CGTCTTGCCATCTTACAGTCTTA	
CAV-com-F2	CGAGTACAGGGTAAGCGAGCTAAA	990bp
CAV-com-R2	TGCTATTCATGCAGCGGACTT	
CAV-com-F3	ACGAGCAACAGTACCCTGCTA	802bp
CAV-com-R3	CTGTACATGCTCCACTCGTT	

**Table 2:** CIAV strains were used as reference strains in this study.

**Strains**	**Country and Time**	**Host**	**Accession No**.	**Whole length**
SDLY08	2008 China	Chicken	FJ172347	2298 bp
3711	2007 Australia	Chicken	EF683159	2279 bp
CAV-6	2014 China	Chicken	KJ728817	2298 bp
CAV-14	2014 China	Chicken	KJ728824	2298 bp
E51057	2000 Japan	Chicken	E51057	2298 bp
SC-MZ	2014 China	Chicken	KM496306	2298 bp
CAT-CA V	2014 China	Chicken	KC414026	2295 bp
CIA V89-69	2013 Korea	Chicken	JF507715	2298 bp
GD-1-12	2012 China	Chicken	JX260426	2298 bp
LF4	2005 China	Chicken	AY839944	2298 bp
HH982173	2006 USA	Chicken	HH982173	2298 bp
AB031296	2000 Japan	Chicken	AB031296	2298 bp
AGV2	2012 China	Human	JQ690762	2316 bp
L14767	1999 USA	Chicken	L14767	2298 bp
3-1	2003 Malaysia	Chicken	AF390038	2298 bp
A48606	1996 USA	Chicken	A48606	2298 bp
CAU66304	1997 UK	Chicken	U66304	2319 bp
clone 33	2002 Germany	Chicken	AJ297684	2298 bp
NC001427	2015 USA	Chicken	NC001427	2319 bp
Cux-1	2008 Netherlands	Chicken	M55918	2319 bp
DI072479	1990 USA	Chicken	DI072479	2298 bp
CAV-4	2014 China	Chicken	KJ728816	2298 bp
CAV-18	2014 China	Chicken	KJ728827	2298 bp
GD-K-12	2013 China	Chicken	KF224935	2298 bp
AH4	2005 China	Chicken	DQ124936	2298 bp
AB119448	2009 Japan	Chicken	AB119448	2298 bp
TR20	1999 Japan	Chicken	AB027470	2298 bp
Cuxhaven	1992 Germany	Chicken	M81223	2298 bp
AB1K	2020 Turkey	Chicken	MT259319	2296 bp
M81223	1993 Germany	Chicken	M81223	2298 bp
CAU65414	1996 Australia	Chicken	CAU65414	2298 bp
A2	2000 Japan	Chicken	AB031296	2298 bp
AF313470	2000 USA	Chicken	AF313470	2294 bp
AF227982	2001 Australia	Chicken	AF227982	2286 bp
AB046590	2001 Japan	Chicken	AB046590	2298 bp
AF475908	2002 China	Chicken	AF475908	2298 bp
clone 34	2002 Germany	Chicken	AJ297685	2297 bp
SMSC-1P60	2003 Malaysia	Chicken	AF390102	2298 bp
SMSC-1	2003 Malaysia	Chicken	AF285882	2298 bp
BD-3	2004 Bangladesh	Chicken	AF395114	2298 bp
C14	2004 China	Chicken	EF176599	2298 bp
SD22	2005 China	Chicken	DQ141673	2298 bp
DQ217401	2005 Malaysia	Chicken	SMSC-1P123	2298 bp
CAE26P4	2007 Netherlands	Chicken	D10068	2298 bp
01-4201	2007 USA	Chicken	DQ991394	2298 bp
Cuxhaven-1	2008 Netherlands	Chicken	M55918	2319 bp
CAECA123	2008 Japan	Chicken	D31965	2319 bp
98D02152	2010 USA	Chicken	AF311892	2298 bp
GXC060821	2012 China	Chicken	JX964755	2292 bp
CAV-10	2014 Argentina	Chicken	KJ872513	2298 bp
KM496307	2014 China	Chicken	SC-MZ42A	2298 bp
Isolate 18	2014 Taiwan	Chicken	KJ728827	2298 bp
SD15	2015 China	Chicken	KX811526	2298 bp
JS2020-PFV	2020 China	Chicken	MW234428	2296 bp
GX1804	2018 China	Chicken	MK484615	2298 bp
SD1510	2016 China	Chicken	KU598851	2298 bp
HN1405	2016 China	Chicken	KU645520	2298 bp

### Fluorescent Quantitative PCR

With the fluorescent quantitative PCR kit produced by TaKaRa Biotechnology (Takara, Dalian, Chian), the sample DNA was detected and verified by the fluorescent quantitative PCR method according to the references (7).

### Dot Blot Hybridization

The samples were tested by PCR combined with dot blot hybridization (18), and the nitrocellulose membrane was purchased from Boehringer Millipore (Merck, Germany).

### Amplification and Sequencing of the Whole Genome of Samples

According to the sequence published in the GenBank, three primers were designed to amplify the sample DNA using DNAStar 6.0 application. The primers are shown in Table 1. The amplification procedure was as follows: 94°C for 5 min; 94°C for 30 s, 55°C for 30 s, and 72°C for 30 s (34 cycles); 72°C for 10 min (35 cycles); and 4°C. The PCR products were identified using 1% agarose gel electrophoresis and stained with ethidium bromide. The bands were purified with an Omega gel recovery and purification kit (Omega Bio-Tek, Norcross, GA, USA), and the recovered target fragments were attached to the pMD18-T vector (Takara, Dalian, Chian). After overnight ligation at 16°C, the ligation products were transformed into competent cells of E. coli DH5α (Takara, Japan), and the positive clones were screened. Finally, the bacterial liquid identified as positive by PCR was sent to Shanghai Bioengineering Co., Ltd. for sequencing.

### Sequence Analysis

The DNA sequences were assembled using DNAStar (version 6.0). Multiple sequence alignment was performed using the Clustal W (BioEdit version 7.0) program, and the comparison of sequence identity was performed using MegAlign software (DNAStar). Phylogenetic analysis was performed using the maximum likelihood (ML) method on RAxML.

## Results

### Results of CIAV Nucleic Acid Test in Vaccines

**Figure 1:**
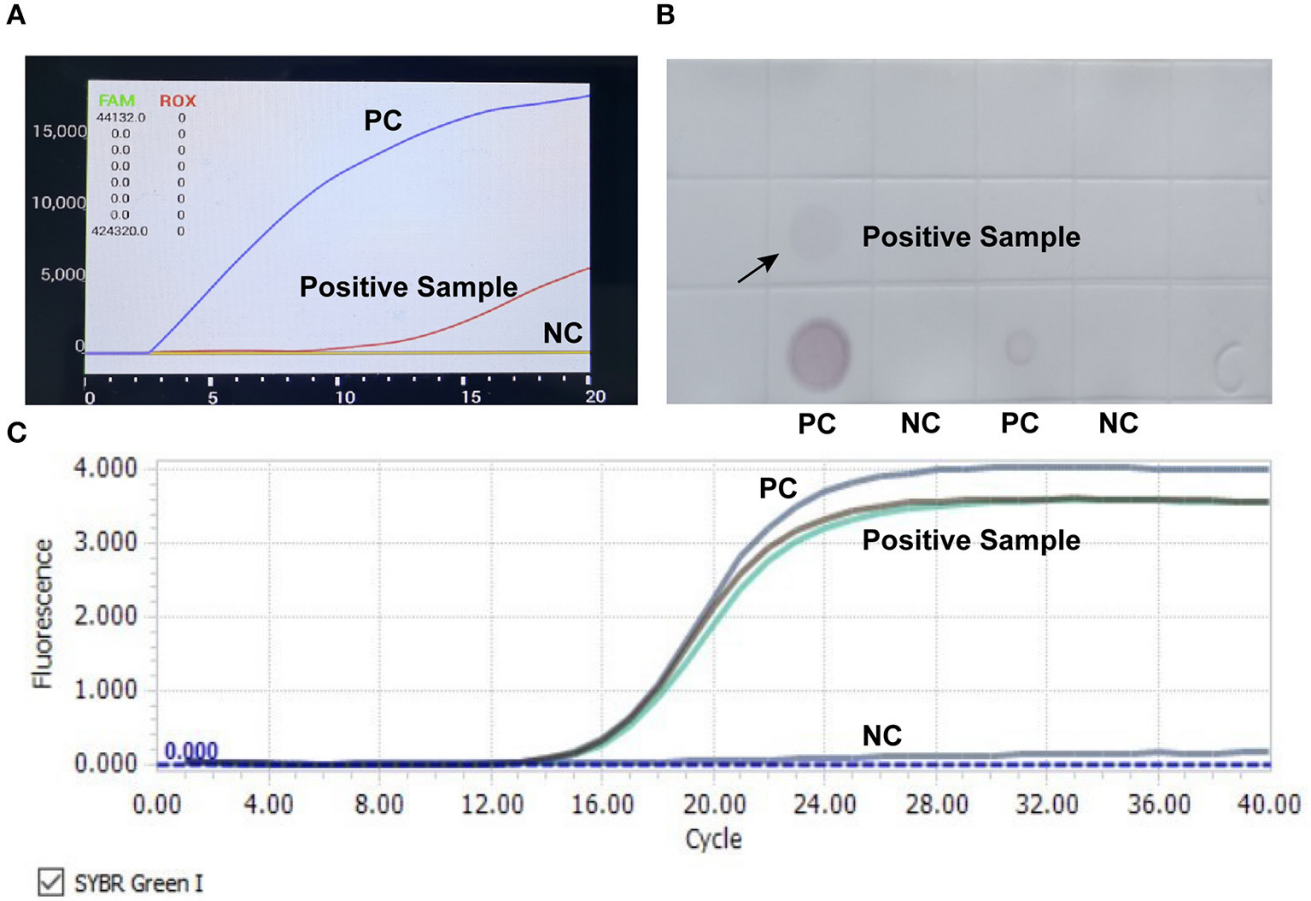
Detection of CIAV nucleic acid in attenuated chicken pox vaccine. RAA rapid assay **(A)**, nucleic acid dot hybridization **(B)**, and fluorescence quantitative PCR assay **(C)** were simultaneously employed to detect CIAV contamination in vaccine samples, and all the methods showed positive results. PC, positive control; NC, negative control.

RAA, qPCR assay, and dot blot were employed to detect CIAV contamination in live vaccines. Results showed that a fowlpox attenuated vaccine sample was determined as CIAV positive in all three methods (Figure 1).

### Whole-Genome Sequencing

**Figure 2:**
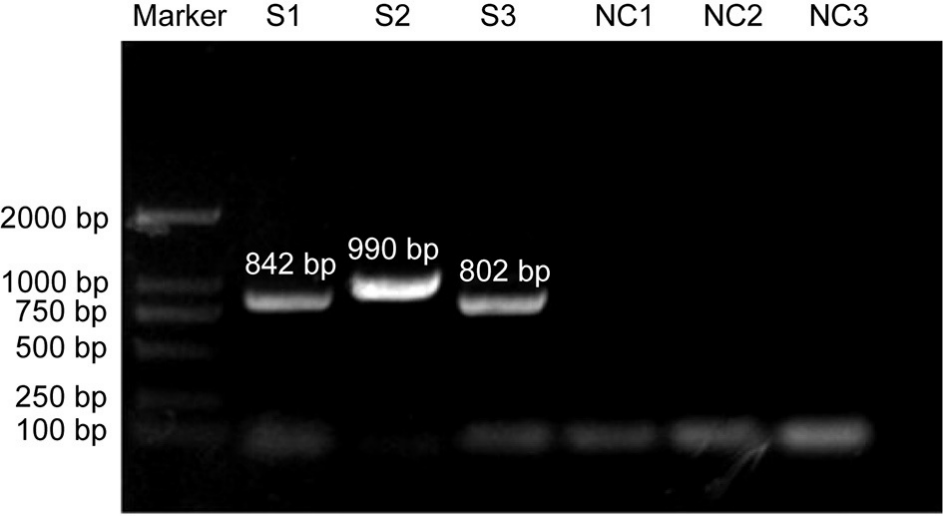
PCR amplication of CIAV in contaminated vaccine using three pairs of primers. S: segment of CIAV; NC: negative control.

To further reveal the molecular characteristics of the CIAV contaminants, the whole genome of this strain was sequenced using three pairs of primers. The electrophoretic analysis confirmed the positive amplification by PCR, and the three segments were 842bp, 990bp and 802bp, respectively (Figure 2), which was consistent with the expected band size. Furthermore, the whole genome of this isolate was 2296 bp, covering three open-reading-frame (ORF), namely VP1, VP2, and VP3.

The lengths of VP1, VP2 and VP3 were 1350 bp, 651 bp and 366 bp, respectively. The isolate was named JS2020-PFV and its sequence was uploaded to the GenBank gene library with the accession number of MW234428.

### Genome Analysis of JS2020-PFV

**Figure 3:**
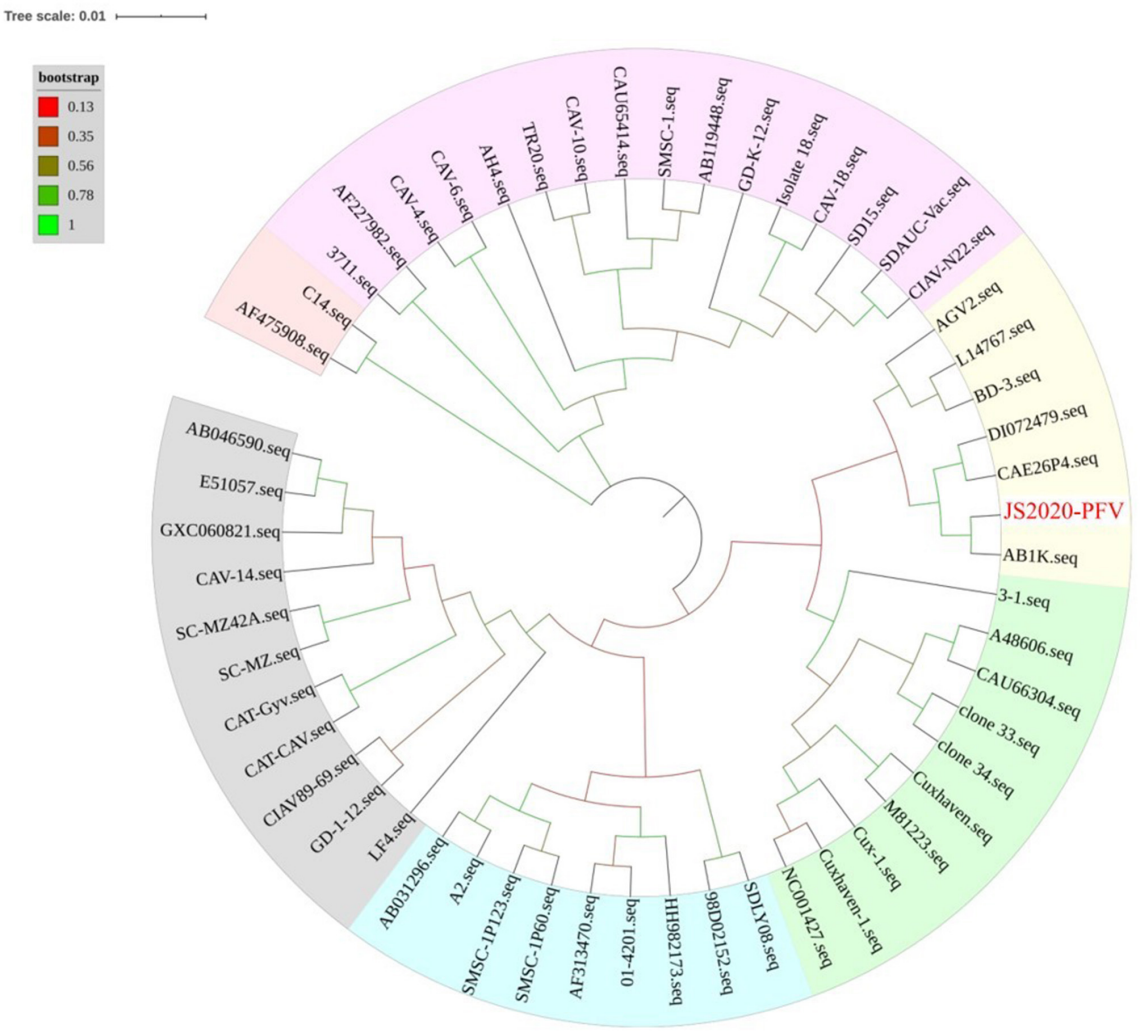
Phylogenetic tree of full-length viral genome of JS2020-PFV and reference strains. The isolated strain in this study has been highlighted in red. The tree was constructed by the maximum likelihood method with 1,000 bootstrap replicates using MEGA 5.0.

The whole genome of the JS2020-PFV strain was compared with that of 56 other CIAV strains. Results showed that JS2020-PFV shared 94.5 to 99.9% of identities with other reference strains. Among them, JS2020-PFV had the highest identity (99%) with the AB1K strain (MT259319) isolated from a chicken farm in Turkey. A phylogenetic tree was constructed based on the whole genome sequence of JS2020-PFV and reference strains, and the results further confirmed the closest evolution relationship between JS2020-PFV and AB1K (Figure 3).

### Analysis of VP1 Gene Variation in JS2020-PFV Strain

The VP1 gene of the JS2020-PFV strain shared 94.0 to 99.9% identities with the reference strains, with the highest identity (99.9%) with AB1K. The genetic evolution relationship between the VP1 gene of the JS2020-PFV strain and reference strains was consistent with the whole genome sequence (Figure 3). Further analysis of the VP1 gene of JS2020-PFV strain revealed that there are two amino acid mutations within which had never been found before, namely threonine at position 89 of VP1 protein changed to lysine and glutamine at position 448 changed to lysine (Table 3).

**Table 3:** Position of mutational VP1 amino acid of JS2020-PFV.

**Strains**	**Position of VP1 amino acid**										
	**89**	**92**	**125**	**139**	**141**	**144**	**157**	**254**	**370**	**447**	**448**
Cux-1	T	G	I	K	Q	D	V	G	S	T	Q
GD-F-1	T	G	L	K	Q	E	M	E	G	S	Q
SDLY08	T	G	I	K	R	E	V	E	G	T	Q
SD15	T	G	I	K	Q	Q	V	E	T	S	P
AB1K	T	D	I	K	Q	E	M	G	S	T	Q
JS2020-PFV	**K**	D	I	K	Q	E	M	G	S	T	**H**

### Difference Analysis of Transcriptional Regulatory Elements in Non-coding Regions

**Figure 4:**
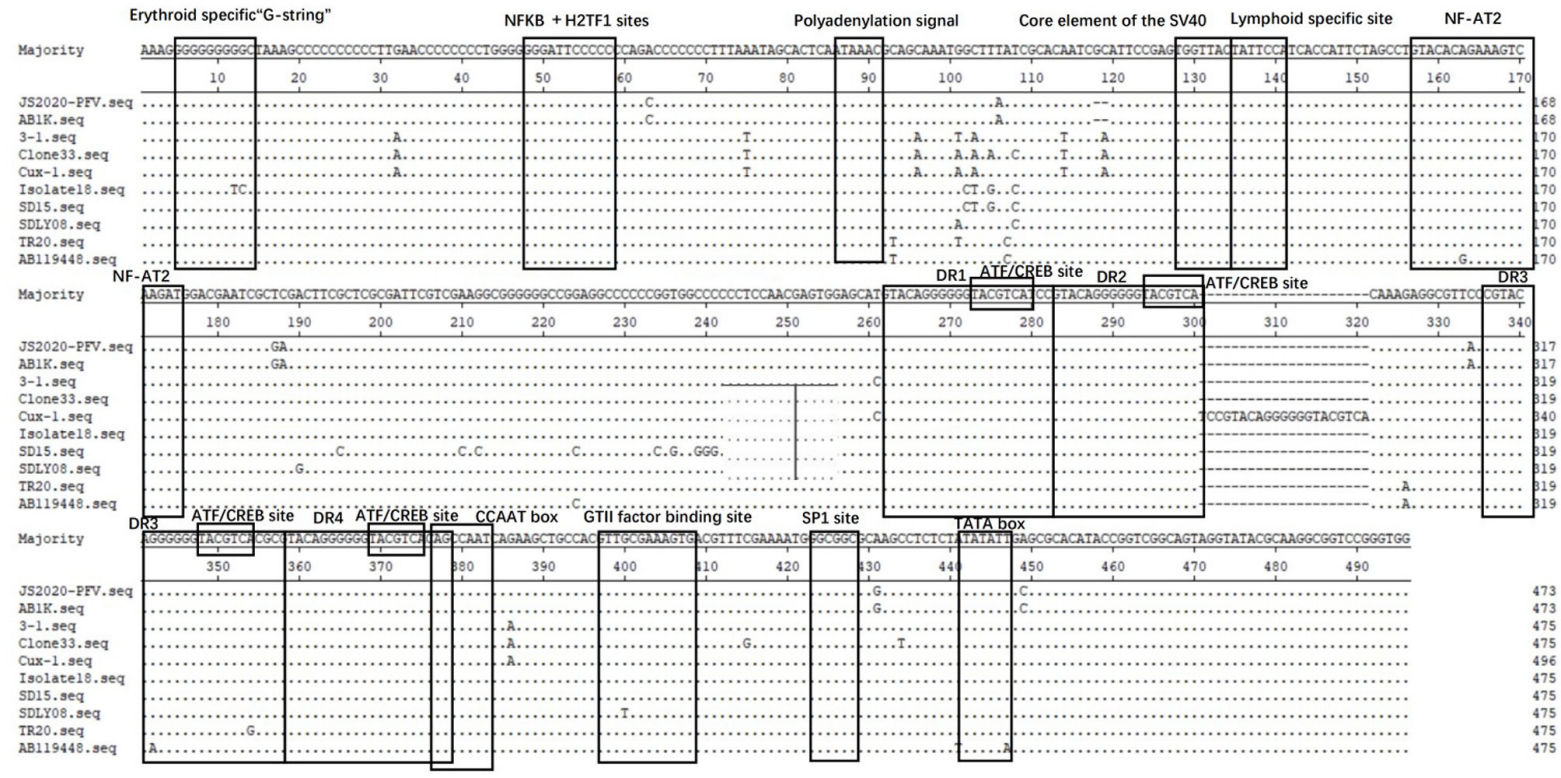
Structural analysis of the non-coding region of JS2020-PFV strain and reference strains. The sequences in black frames are the motifs of transcriptional regulatory elements in this study.

The Clustal W method was used for multiple alignment analysis of the non-coding regions of JS2020-PFV with 9 reference strains. Compared with the reference strains, the motif in non-coding regions of CIAV was highly conserved (Figure 4). Softberry's Nsite online service analysis showed that four CREB sites associated with viral apoptotic capacity were also conserved in the new isolate. The JS2020-PFV and most of the reference strains all had four DR regions in the non-coding region, except for one additional DR region in the CUX-1 strain.

## Discussion

Over the past two decades, many live vaccines have been proved to be contaminated with exogenous viruses, including avian leukosis virus (19), fowl adenovirus (20), reticuloendotheliosis virus (26), CIAV (6) and others. All these viruses share the common feature of being able to vertically propagate through the chicken embryo, suggesting such contamination is mainly caused by the use of SPF chicken embryos infected with exogenous viruses during vaccine production. Several studies have reported the infection of the above viruses in SPF chicken farms, which further confirmed the above hypothesis (21). The use of contaminated vaccines will not only cause the spread of the virus but also cause serious clinical symptoms, especially vaccines contaminated with CIAV that induce strong immunosuppression. Previous studies demonstrated that the co-infection with CIAV can significantly improve the pathogenicity of the LaSota strain in NDV vaccines, and decrease the antibody titer, which makes the vaccinated chickens vulnerable to wild NDV strains (7). Therefore, monitoring the exogenous virus contamination in live vaccination is of great significance for poultry farms.

In this study, RAA assay, the traditional qPCR and dot blot were used at the same time to screen the potential contamination of CIAV in live vaccines. Results found that one fowl pox vaccine was positive for CIAV in all these three methods, indicating that the detection results were true and reliable. It should be noted that RAA completed the whole detection process in only 15 min, while qPCR and dot blot took 1.5 and 14 h respectively. Now, there are also several handheld devices for RAA. Therefore, the RAA method is more suitable for monitoring vaccine contamination in farms because of its flexibility and convenience.

Furthermore, the whole genome sequence of CIAV was sequenced and named JS2020-PFV. It was found that JS2020-PFV had extremely high identities (99%) and the closest evolution relationship with the AB1K strain (MT259319) that was isolated from a chicken farm in Turkey. Besides, JS2020-PFV shared a close relationship with American isolate (DI072479) and Netherland isolate (CAE26P4), while it was relatively unrelated to Chinese isolates, including SDLY18 (FJ172347), GD-K-12 (KF224935) and SD15 (KX811526). These results are consistent with the fact that the vaccine was imported from a foreign company, suggesting that the transnational trade of vaccines may be an important way for some strains to spread across countries and regions.

The amino acid positions of VP1 protein are closely related to CIAV pathogenicity and cell proliferation (5). Previous studies have shown that amino acid positions 139 and 144 on VP1 were associated with the efficiency of virus proliferation and transmission in MSB1 cells (22). The amino acid positions 139 and 144 on the VP1 protein of JS2020-PFV are lysine (K) and glutamic acid (E), which are consistent with the strain with high replication ability (22). Besides, glutamine (Q) at position 394 was associated with higher pathogenicity, while histidine (H) at position 394 was related to lower pathogenicity (22). In this study, the amino acid at position 394 of VP1 protein of JS2020-PFV is glutamine (Q), suggesting that this strain may also be highly pathogenic. There are more than ten motifs related to transcriptional regulation in the non-coding region of CAV, which are closely related to viral replication and transcriptional regulation. After comparing and analyzing the non-coding regions of the selected strains in this paper, it was found that these motifs were very conservative. We speculate that these related motifs may be necessary for the replication of the virus itself.

Overall, CIAV contamination was detected from a fowlpox vaccine, and genome analysis suggested that it may be a highly pathogenic strain, which reminded us to pay close attention to the possible contamination of exogenous virus in live poultry vaccine. To prevent the recurrence of this phenomenon, SPF chicken farms, vaccine factories and farmers all should take reasonable countermeasures. First of all, SPF chicken farms should strengthen the daily monitoring of viruses, regularly detect the viruses with vertical transmission ability that have been reported in contaminated vaccines, and strictly eradicate their breeding chicken flocks. After purchasing chicken embryos, vaccine factories should also conduct viral detections of chicken embryos to eliminate the possibility of exogenous virus infection. In the last link, farmers should conduct a spot check on each batch of vaccines before using, to eliminate the possibility of exogenous virus contamination. The convenient and fast RAA method applies to the above three links, which is very conducive for on-site detection.

## Data Availability Statement

The datasets presented in this study can be found in online repositories. The names of the repository/repositories and accession number(s) can be found below: https://www.ncbi.nlm.nih.gov/, MW234428.

## Author Contributions

LC and QS conceived and performed the experiments, analyzed the data, and drafted the manuscript. PZ supervised the project and edited the manuscript. YL, JW, YZ, SC, and YW performed part of the experiments. All authors contributed to the article and approved the submitted version.

## Funding

This work was supported by the National Key Research and Development Program of China (2018YFD0500106).

## Conflict of Interest

The authors declare that the research was conducted in the absence of any commercial or financial relationships that could be construed as a potential conflict of interest.

## Publisher's Note

All claims expressed in this article are solely those of the authors and do not necessarily represent those of their affiliated organizations, or those of the publisher, the editors and the reviewers. Any product that may be evaluated in this article, or claim that may be made by its manufacturer, is not guaranteed or endorsed by the publisher.
